# Bacteriophage tailspike protein based assay to monitor phase variable glucosylations in *Salmonella* O-antigens

**DOI:** 10.1186/s12866-016-0826-0

**Published:** 2016-09-07

**Authors:** Andreas Schmidt, Wolfgang Rabsch, Nina K. Broeker, Stefanie Barbirz

**Affiliations:** 1 Physikalische Biochemie, Universität Potsdam, Karl-Liebknecht-Str. 24-25, Golm, 14476 Germany; 2 National Reference Centre for Salmonella and other Bacterial Enterics, Robert Koch Institute, Burgstraße 37, Wernigerode, 38855 Germany

**Keywords:** *Salmonella* Typhimurium, O-antigen, Tailspike protein, Bacteriophage, Phase variation, O-serotyping, Flow cytometry

## Abstract

**Background:**

Non-typhoid *Salmonella* Typhimurium (*S.* Typhimurium) accounts for a high number of registered salmonellosis cases, and O-serotyping is one important tool for monitoring epidemiology and spread of the disease. Moreover, variations in glucosylated O-antigens are related to immunogenicity and spread in the host. However, classical autoagglutination tests combined with the analysis of specific genetic markers cannot always reliably register phase variable glucose modifications expressed on *Salmonella* O-antigens and additional tools to monitor O-antigen glucosylation phenotypes of *S.* Typhimurium would be desirable.

**Results:**

We developed a test for the phase variable O-antigen glucosylation state of *S.* Typhimurium using the tailspike proteins (TSP) of *Salmonella* phages 9NA and P22. We used this ELISA like tailspike adsorption (ELITA) assay to analyze a library of 44 *Salmonella* strains. ELITA was successful in discriminating strains that carried glucose 1-6 linked to the galactose of O-polysaccharide backbone (serotype O1) from non-glucosylated strains. This was shown by O-antigen compositional analyses of the respective strains with mass spectrometry and capillary electrophoresis. The ELITA test worked rapidly in a microtiter plate format and was highly O-antigen specific. Moreover, TSP as probes could also detect glucosylated strains in flow cytometry and distinguish multiphasic cultures differing in their glucosylation state.

**Conclusions:**

Tailspike proteins contain large binding sites with precisely defined specificities and are therefore promising tools to be included in serotyping procedures as rapid serotyping agents in addition to antibodies. In this study, 9NA and P22TSP as probes could specifically distinguish glucosylation phenotypes of *Salmonella* on microtiter plate assays and in flow cytometry. This opens the possibility for flow sorting of cell populations for subsequent genetic analyses or for monitoring phase variations during large scale O-antigen preparations necessary for vaccine production.

**Electronic supplementary material:**

The online version of this article (doi:10.1186/s12866-016-0826-0) contains supplementary material, which is available to authorized users.

## Background

Antimicrobial strategies usually focus on bacterial surfaces, for example in classic antibiotics that interfere with cell wall synthesis pathways [1], and also in phage therapy approaches that target the outer membrane for penetration [2]. Moreover, classical immunological diagnostics rely on the recognition of specific surface compounds by antibodies which are described for *Salmonellae* in the Kauffmann-White-Le Minor typing scheme [3]. It defines, amongst others, the O-antigen, a repetitive polysaccharide of highly diverse carbohydrate composition [4]. In *Salmonellae*, O-antigens often vary in their glucosylation states [5]. *Salmonellae* contain up to four *gtr* operons for glucose transfer controlled by epigenetic phase variation [6] and of bacteriophage origin, adding an additional level of complexity to O-antigen structure which is only partly reflected by the Kauffmann-White-Le Minor scheme [7]. *S.* Typhimurium has the O-serotype O4, O12 and is one of the most commonly isolated strains in non-typhoid Salmonellosis infections. Furthermore, as a consequence of phase variation glucose can be 1-4 linked (serotype O12-2) or 1-6 linked (serotype O1) to the galactose of O-polysaccharide backbone [6–9]. In addition, O-antigen acetylation can occur, which defines the serotype O4,(5) [9]. Serotypes are usually classified by slide agglutination tests with antibodies in combination with analysis of genetic markers [10], but these tests do not always reliably reflect the highly variable glucosylation state of the *S.* Typhimurium O-antigen. For example, phase variable O-antigen glucosylations may influence the evasion of *Salmonella* from complement [11] or can occur only upon exposure to eukaryotic cells, whereas they may be absent in bacteriologic media [5]. Moreover, glucosylations also protect *Salmonella* from bacteriophage infection [12] and regulate prophage associated O-antigen variability [13].

Bacteriophage tailspike proteins (TSP) of lipopolysaccharide (LPS) specific phages recognize and cleave the O-antigen to position the phage towards a secondary membrane receptor during infection [14–16]. They possess large carbohydrate interaction sites that bind O-antigen with high specificity [17–19]. TSP are highly thermostable and protease resistant [20]. TSP of bacteriophage 9NA and P22 recognize *S.* Typhimurium and have endorhamnosidase activity to cleave the O-antigen [16, 21]. In this work we present a methodology using 9NA and P22TSP as probes for the rapid detection of O-antigen glucosylation in *S.* Typhimurium both on microtiter plates and in flow cytometry.

## Results

### ELISA-like tailspike adsorption (ELITA) assay

**Fig. 1:**
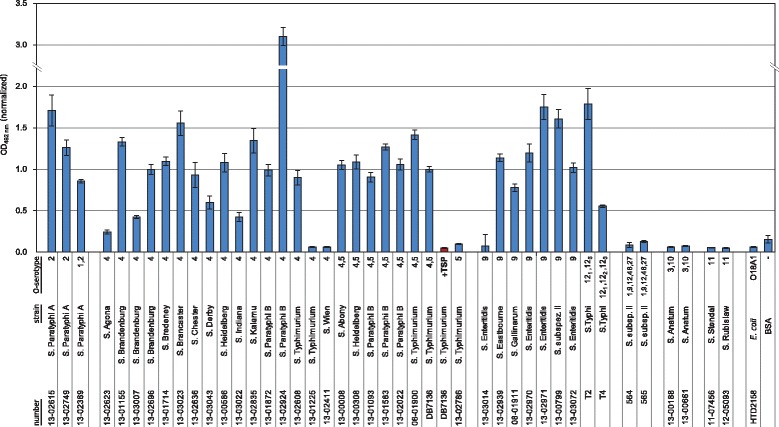
Analysis of *Salmonella* strains with ELISA-like tailspike adsorption (ELITA) assay using *Strep*-tag®II-labelled 9NATSP as a probe. One sample shows strain DB7136 incubated with 9NATSP wild type prior to ELITA (*red bar*). Strain *E. coli* HTD2158 [38] and bovine serum albumin (BSA) were used as a negative control. Numbers refer to the Wernigerode collection (*cf.* Table 3). Error bars represent standard deviation from four replicate experiments

To rapidly select for bacterial cultures binding to bacteriophage tailspike proteins (TSP) we developed a microtiter-plate based assay. For this, the bacteria were adsorbed to the surfaces of the wells. We then used *Strep*-tag®II-labelled 9NATSP to probe binding to 44 *Salmonella* strains of the Wernigerode collection and read out the signal with a *Strep*-Tactin® labeled horseradish peroxidase. The resulting ELISA-like tailspike adsorption (ELITA) procedure was highly specific (Fig. 1). 9NATSP recognized strains of O-serogroups O2, O4,(5) and O9. These are the serogroups found in the host range of bacteriophage 9NA [21]. By contrast, no signals were observed for non-host strains and for an *E. coli* strain defined as the false positive control. The host strain *S.* Typhimurium DB7136 used to propagate bacteriophage 9NA was used to normalize the binding signal and served as false negative control. The test was repeated four times and standard deviation from the independent experiments was less than 10 %. In the ELITA test we used a TSP that was mutated to inactivate its enzymatic cleavage of the O-polysaccharide in order to obtain a stable binding signal [16]. If we pre-incubated the bacteria with an enzymatically active TSP, no binding was detectable with the *Strep*-tag®II-labelled 9NATSP probe afterwards (Fig. 1). This further confirmed that the 9NATSP binding signal in the ELITA was clearly O-antigen specific. However, within the subset of binding strains, 9NATSP showed signal intensities varying from strain to strain. We purified several LPS from strains with different signals in the ELITA assay and did not observe varying chain lengths which might account for different numbers of TSP binding sites and therefore varying ELITA signals (data not shown). Rather, it is plausible that the ELITA test reflected the heterogeneous adsorption behavior of different strains to the microtiter-plate surface.

### Analysis of O-antigen composition of 9NATSP binding Salmonella strains

**Fig. 2:**
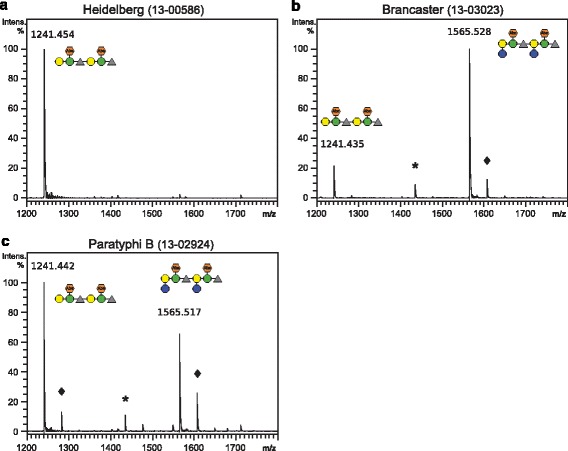
MALDI-MS of the two repeat unit fraction oligosaccharides obtained from 9NATSP cleavage of polysaccharide isolated from **a** *S.* Heidelberg 586, **b** *S.* Brancaster, and **c** *S.* Paratyphi B 2924. Putative oligosaccharide structures are given in CFG notation [42] with abequose symbolized as red hexagon. Asterisks mark peaks that lost abequose, diamonds mark acetylated peaks, respectively. All theoretical and experimental masses are given in Table 1

About half of the strains tested were of O-serogroup O4 or O4,(5). These O-antigens have the trisaccharide backbone structure α-d-Man*p*-1-4-α-l-Rha*p*-1-3-α-d-Gal*p*-α-1-2 [22]. The didesoxyhexose abequose α-1-3-linked to mannose determines the serotype O4, if the abequose is acetylated, the serotype O4,(5) results [9]. We chose several of these strains that showed similar binding signals in ELITA with 9NATSP and examined the molecular composition of the O-antigen. For this, we analyzed oligosaccharides purified from 9NATSP-O-polysaccharide digests with MALDI-MS (Fig. 2 and Table 1). The mass spectra showed peaks corresponding to fragments of two O-antigen repeat units (2RU) as main digestion products in all samples in agreement with precedent studies on O4 lipopolysaccharides [16]. For several strains acetylations were detected. Additionally, some strains showed peaks at higher masses corresponding to 2RU fragments with two additional hexoses, for example in case of *S*. Brancaster (Fig. 2b). The analysis of the monosaccharide composition of *S.* Brancaster O-polysaccharide with high performance anion exchange with pulsed amperometric detection (HPAEC-PAD) confirmed the presence of glucose in the O-antigen of this strain (Additional file 1: Figure S1). Glucosylated oligosaccharides were also detected with polysaccharide from *S*. Paratyhi B 2924, from *S.* Kalamu and *S.* Heidelberg 308. By contrast, polysaccharide from strain *S.* Heidelberg 586 only showed non-glycosylated oligosaccharides. From this we conclude that 9NATSP tolerates a glucosylated O-antigen.

**Table 1:** Monoisotopic calculated and experimental mass analyses from MALDI-MS of two repeat unit (2RU) oligosaccharides obtained from O-antigen polysaccharide enzymatic cleavage with 9NATSP

Oligosaccharide composition^a^	[M + Na]^+^_calc_/Da	[M + Na]^+^_exp_/Da		
		*Salmonella* strain		
		Heidelberg 586	Brancaster	Paratyphi B
2 RU	1241.45	1241.45	1241.44	1241.44
2 RU + 2Glc–Abe^b^	1435.41	*n.d.* ^c^	1435.47	1435.44
2 RU + 2Glc	1565.56	*n.d.* ^c^	1565.53	1565.52
2 RU + 2Glc + Ac	1607.57	*n.d.* ^c^	1607.53	1607.52

^a^two repeat units of basic repeat α-d-Gal*p*-1-2-α-d-Man*p*(−α-3-1-Abe*p*)-1-4-α-l-Rha*p*-1-3- as produced by TSP cleavage [44], Abe: Abequose, Ac: Acetylation

^b^without Abequose

^c^not detected

### Comparison of binding properties of two Salmonella phage TSP with similar host range

**Fig. 3:**
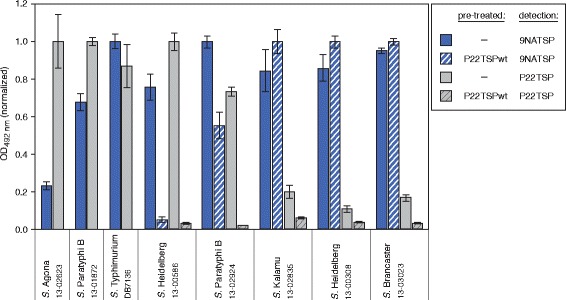
ELISA-like tailspike adsorption (ELITA) assay with *Salmonella* strains of serogroup O4,(5) using 9NATSP (*blue*) or P22TSP (*gray*) as probes. Crosshatched bars represent data obtained after pre-treatment of immobilized bacteria with 50 μg/ml of catalytically active P22TSP to destroy the O-antigen receptor. Signals were normalized individually for each single strain; error bars represent standard deviation from four replicate experiments.

**Fig. 4:**
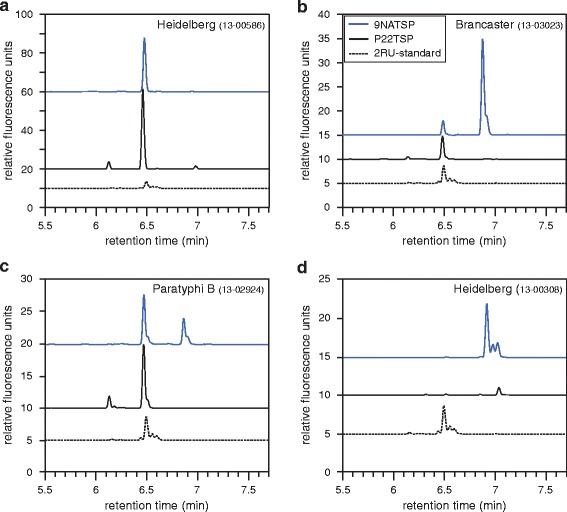
Analysis of oligosaccharide mixtures obtained from *Salmonella* LPS after TSP enzymatic cleavage with capillary electrophoresis and laser-induced fluorescence detection (CE-LIF). Profiles from LPS of *S.* Heidelberg 586 **a**, *S.* Brancaster **b**, *S.* Paratyphi B 2924 **c**, *S.* Heidelberg 308 **d** shown after treatment with 9NATSP (*blue*) or P22TSP (*black*). The CE-LIF profile of a 2RU standard shown as dashed line

The 44 *Salmonella* strains analyzed with the 9NATSP probe were subsequently tested in the ELITA assay using a TSP probe from bacteriophage P22 (see Additional file 2: Figure S2 for the full data set). P22TSP is structurally very similar to 9NATSP because both phages 9NA and P22 use their TSP as essential infection organelles for specific host adsorption on a similar host range [16]. In the ELITA assay, P22TSP, like 9NATSP, clearly distinguished all host from non-host strains. However, within a single strain P22TSP and 9NATSP markedly differed in their binding signals. This was distinctly visible in the O4,(5) subgroup, where P22TSP showed higher signals for *S.* Heidelberg 586, *S.* Paratyphi B 1872 and *S.* Agona (Fig. 3). By contrast, P22TSP displayed markedly reduced binding signals for the strains *S.* Brancaster, *S.* Kalamu, *S.* Heidelberg 308 and *S.* Paratyphi B 2924. These strains all contain glucose in their O-antigens. They were not only impaired in P22TSP binding but also in O-polysaccharide cleavage by P22TSP (Fig. 3). If they were pre-treated with enzymatically active P22TSP, binding was still detectable after this with the 9NATSP probe, showing that the O-antigen had remained intact. This data is in agreement with the fact that P22TSP and 9NATSP differ in their ability to recognize and cleave glucose containing O4-polysaccharides [21]. To confirm this we analyzed the products obtained from TSP-mediated cleavage of LPS preparations isolated from different O4,(5) *Salmonella* strains with capillary electrophoresis (CE) (Fig. 4). Here, the main 2RU cleavage products eluted at later retention time when they contained glucose. 9NATSP and P22TSP both produced octasaccharides from the non-glucosylated strain *S.* Heidelberg 586. From all other strains that were glucosylated, P22TSP only produced non-glucosylated octasaccharides. By contrast, analyzing 9NATSP LPS cleavage products in CE showed 2RU products containing 89 % glucose in *S.* Brancaster, 39 % in *S.* Paratyphi B 2924 and 100 % in *S.* Heidelberg 308, respectively.

### Flow cytometry analysis of 9NATSP and P22TSP binding to Salmonella

**Fig. 5:**
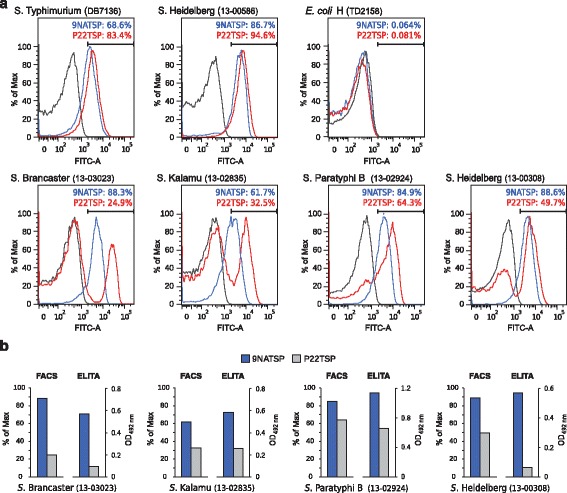
**a** Flow cytometry histograms of a representative set of *Salmonella* strains from serogroup O4,(5) probed with fluorescently tagged TSP. **b** Qualitative comparison of signal intensities from ELITA assay and flow cytometry

O-antigen glucosylation can occur as a phenotype during phase variation in *Salmonella* [6]. We analyzed bacterial cultures with flow cytometry for the occurrence of heterogenous populations [23]. Moreover, the flow cytometry experiment served as a control to exclude any matrix effects in the microtiter plate based ELITA. We incubated bacteria with *Strep*-tag®II-labelled TSP, stained bound TSP with a fluorescent *Strep*-Tactin®-Chromeo488 conjugate and subjected the bacteria to flow cytometry. Binding efficiency was measured as the load of TSP per bacterial particle, as deduced from the increase of Chromeo488 fluorescence (see Additional file 3: Figure S3 for the full data sets). 9NA and P22TSP bound to 69 and 83 % of the cells of the reference strain *S*. Typhimurium DB7136, respectively, but not to the *E. coli* control (Fig. 5). Both TSP also bound well to the majority of cells of the non-glycosylated strain *S.* Heidelberg 586 which contained even more binding cells than the reference (86 and 94 %, respectively). By contrast, the glucose containing strains *S.* Brancaster, *S.* Kalamu and *S.* Paratyphi B 2924 displayed reduced binding with P22TSP compared to the signals obtained with 9NATSP, in qualitative agreement with results obtained in the ELITA test (Fig. 5). Apparently, P22TSP only bound to the subpopulations of non-glycosylated cells whereas another subpopulation remained unbound. This increased the TSP to cell ratio and resulted in higher signal intensities for the binding population. We conclude that P22TSP, in contrast to 9NATSP could distinguish glucosylated from non-glycosylated subpopulations in these strains. Interestingly, *S.* Heidelberg 308 showed about 50 % binding to P22TSP and 90 % binding to 9NATSP in flow cytometry. This is contradictory, as this strain was found glucosylated and resistant to P22TSP cleavage (see above). Moreover, *S.* Heidelberg 308 only showed weak binding in the ELITA test. 2RU fragments produced from *S.* Heidelberg 308 by 9NATSP O-antigen migrated in CE as glucosylated oligosaccharides with additional minor peaks indicating acetylated species. We can only speculate at this point, that cells at mid-logarithmic phase as analyzed in flow cytometry displayed smaller amounts of acetyl or glucosyl groups on their surface than those grown to stationary phase used for LPS preparations and in ELITA. In the flow cytometry experiment several strains tended to aggregate in the presence of TSP but not without TSP, as deduced from the size of detected particles (Additional file 3: Figure S3). This is probably due to the multivalency of the detection system with three *Strep*-tags®II per TSP and four binding sites on the *Strep*-Tactin®-Chromeo488 conjugate which results in cross linking of bacteria [24]. Accordingly, no aggregation was observed when adding TSP without tags (data not shown).

### Bacteriophage infection assays

Binding of a TSP to the bacterial surface is an initial step in the bacteriophage infection cycle [15, 16]. Therefore we tested whether TSP binding observed in the ELITA assay or in flow cytometry correlated with infectivity of bacteriophages 9NA and P22 on the respective strains. We determined efficiency of plating (EOP) in soft agar layers on a subset of *Salmonella* strains belonging to the serogroup O4,(5) (Table 2). Both phages displayed similar infectivity on the reference host strain DB7136. Moreover, phage 9NA could infect *S.* Paratyphi B 2924, here 9NATSP also was a very good binder (*cf.* Fig. 3). The tailspike of phage P22 was a good binder on *S.* Heidelberg 586, accordingly the phage could produce a small amount of plaques on this strain (EOP < 0.1 %). All other strains were resistant (EOP < 0.001 %) to both phages, notwithstanding that 9NATSP had shown good binding signals on all, and P22TSP on two of the strains tested. Apparently, more criteria must lead to successful phage infection than just TSP recognition of the right O-serotype polysaccharide. However, comparing phage plating with TSP binding experiments, TSP binding seems at least necessary albeit not sufficient for successful *Salmonella* infection by bacteriophages 9NA and P22.

**Table 2:** Efficiency of plating (EOP) of bacteriophages P22 and 9NA on *Salmonella* of serogroup O4,(5)

*Salmonella* strain	9NA		P22	
	*EOP* ^a^	*pfu* ^b^/ml^−1^	*EOP*	*pfu*/ml^−1^
Typhimurium DB7136	1	1.80 × 10^12^	1	5.00 × 10^11^
Paratyphi B 2924	0.16	2.81 × 10^11^	-	-
Brancaster	-	-	-	-
Kalamu	1.70 × 10^−6^	3.06 × 10^6^	-	-
Heidelberg 13-00586	-	-	1.14 × 10^−3^	5.69 × 10^8^
Heidelberg 13-00308	-	-	-	-

^a^determined relative to *S*. Typhimurium DB7136

^b^number of plaque forming units (pfu) detected

## Discussion

**Fig. 6:**
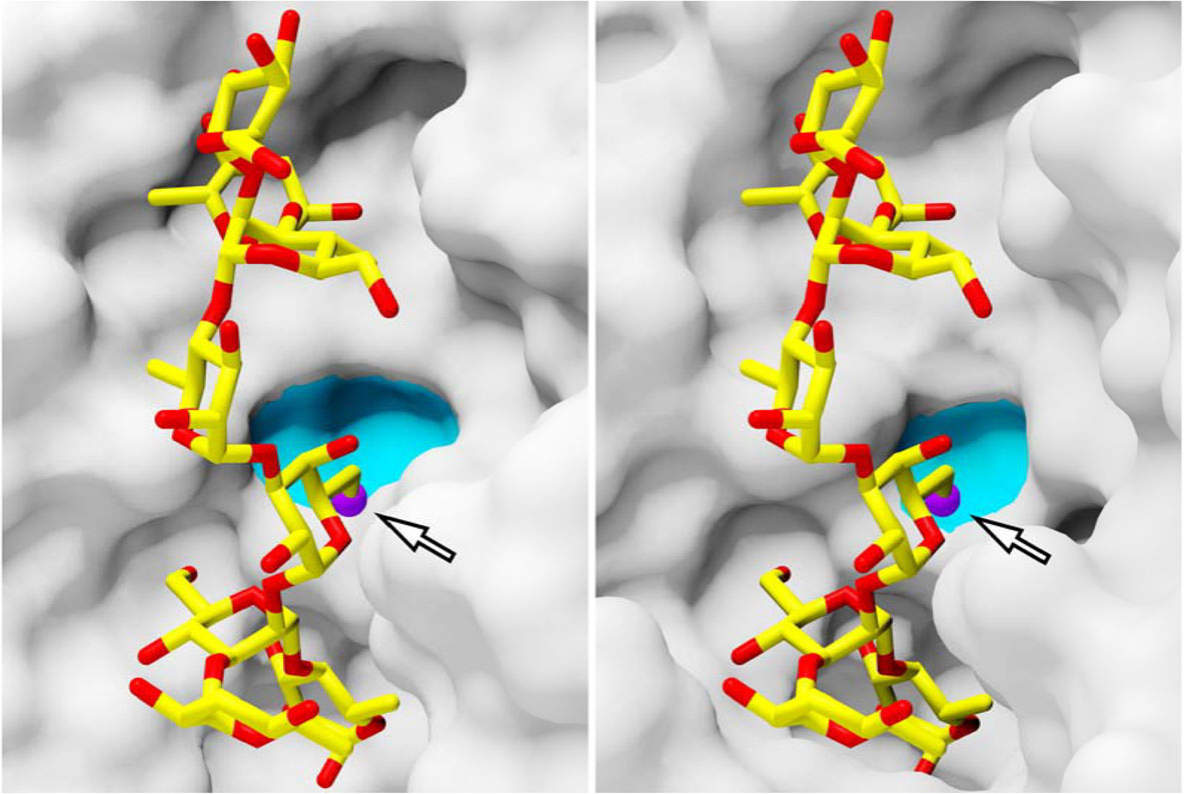
Comparison of 9NATSP and P22TSP O-antigen binding sites. The O6 of galactose pointing to the protein surface is shown in magenta (*see arrow*), a putative glucose binding groove in cyan. Right: Crystal structure of P22TSP with 2RU oligosaccharide (pdb: 1tyx) [26]. Left: Crystal structure of 9NATSP (pdb: 3riq) [16]. The 2RU oligosaccharide was positioned in the binding site after 3D alignment with P22TSP (rmsd 2.54 Å) using the CEALIGN algorithm implemented in PyMOL [43]

In the present study, TSP from siphovirus 9NA and podovirus P22 were used to monitor the glucosylation state of the *Salmonella* O-antigen of serotype O4. We propose that in the ELITA test, both TSP could distinguish the glucosylation state of strains due to their different specificities. P22TSP could not bind to 1-6 glucosylated O-antigen, these serogroup O1 strains carry the bacteriophage P22 lysogen, the glucosylation of which prevents phage P22 from infection [25]. By contrast, 9NATSP bound to these strains, which implies that it can specifically bind 1-6 linked glucose. Comparing the O-antigen binding sites of 9NA and P22TSP we propose that in the P22TSP binding site, glucose at this position would probably not fit into the pocket (Fig. 6). By contrast, the binding site of 9NATSP contains a large surface cavity which could probably accommodate 1-6 linked glucose. This further corroborates that 9NATSP is specific for serogroup O1 whereas P22TSP cannot recognize the 1-6 glucose modification [21]. We also found O4 strains where 9NATSP was a weak binder while P22TSP showed a high signal, i.e. for *S.* Agona, *S.* Brandenburg, *S.* Chester or *S.* Derby (Additional file 2: Figure S2). Although we have not analyzed the glucose content in O-antigens of these strains we propose that they carry 1-4 glucosylations (serotype O12-2) because analysis of phage endorhamnosidase activities had shown earlier that phage 9NA in contrast to phage P22 was unable to produce oligosaccharides from O12-2 strains [21]. This was further supported by an ELITA test on *S*. Typhi, where P22TSP showed good binding on a O12-2 typed strain whereas 9NATSP showed a weak signal on this strain (Additional file 2: Figure S2). P22TSP can bind to O12-2 O-antigens as shown by crystal structure analysis [26]. The overlay of this structure with the 9NATSP binding site could not show why 9NATSP does not tolerate the 1-4 glucosylation and we are yet to solve a crystal structure to analyze why the architecture of the 9NATSP O-antigen interaction site prevents binding of O12-2 O-antigens. Taken together, P22TSP and 9NATSP are thus useful probes to distinguish O1 from non-O1 *Salmonella* serogroup O4 strains when employed in a comparative ELITA test (*cf.* Fig. 3). Moreover, we suggest that they could be employed to distinguish O12-2 positive from O12-2 negative strains, although the molecular details for the lacking interaction with 9NATSP remain to be elucidated.

In the ELITA assay also weak signals occurred for strains that had a glucosylation pattern prohibiting binding to the respective TSP probe. This indicated that heterogeneous cell populations were present on the microtiter plate. Effects related to surface adhesion of bacteria on the plate can be excluded as relative binding efficiencies were similar for the same strains in flow cytometry (*cf.* Fig. 5). More importantly, flow cytometry using 9NA and P22TSP as probes confirmed the presence of heterogeneous populations in the investigated cultures of serotype O1. In these cases, the size of the populations detected with P22TSP correlated well with the proportions of non-glucosylated O-antigen measured in CE-LIF. 9NA and P22TSP are therefore very interesting tools in flow cytometry to monitor glucosylation phenotypes of *Salmonella*. For example they could be used to separate cell populations in a flow sorter and further analyze them by sequencing, with PCR techniques [27] or for studying phase variations in succeeding cell generations. Monitoring phase variations is also important for O-antigen structural analysis or to obtain defined precursors for vaccine production. Here, the glucosylation state is closely related to the immunogenicity of the O-antigen [28]. For the preparation of sufficient material, cells have to be grown to high densities [29]. Flow cytometry with TSP probes could be used here to rapidly assess the glucosylation state of cultures. For strain Heidelberg 308 we observed these differences depending on the time of growth. Whereas overnight cultures as used for O-antigen preparation were fully glucosylated and resistant to P22TSP cleavage, mid-log phase cultures analyzed by flow cytometry using the P22TSP probe showed a binding population indicating non-glucosylated species.

Recognition of defined molecular structures also distinguishes TSP from whole phage typing procedures. Type phages usually are not purely O-antigen specific but also require protein or core LPS receptors for broad specificity. Here, successful infection and replication are a consequence of the multitude of recognition, membrane penetration, replication and lysis events that bacteriophages pass through during their life cycle. This makes phage typing a specific tool for classification of *Salmonella* strains as plaque formation is tightly coupled to the biochemical state of the bacterial cell [30]. However, phage replication may fail due to bacterial defense mechanisms like restriction-modification systems [31], abortive infection [32] or the CRISPR-CAS-system [33]. Phages P22 and 9NA use O-antigen as a primary receptor, but other membrane receptors are also necessary for infection [14, 16]. Their tailspikes’ binding and endorhamnosidase function alone may therefore not be sufficient for infection as shown in this work and in earlier studies [21]. However, we observed a positive correlation between TSP O-antigen binding and plaque formation for bacteriophages 9NA and P22 stressing that O-antigen adsorption is necessary for infection. Heterogeneous cell populations with varying O-antigen structure might also affect plating efficiencies. This was observed with phage 9NA that probably only infected one subpopulation in the biphasic strain of *S.* Paratyphi B 2924 (*cf.* Table 2). However, the bacterial phenotypes resulting from phase variation may include many more changes on the cellular level beyond O-antigen modification that could interfere with plaque formation.

Taken together, their high O-antigen specificity makes TSP interesting serotyping tools, especially when compared to antibodies. Epitopes recognized by antibodies usually are small and often only span less than one biological repeat of the O-antigen [34]. As a result, typing antibodies cannot always detect O-antigen heterogeneities, in contrast to TSP that have large binding sites spanning at least a complete repeat unit. Moreover, typing antibodies often show batch to batch variations and the agglutination procedure only yields positive or negative results, but no further information on heterogeneous phenotypes in a culture. These drawbacks could be overcome in future by developing a set of TSP with specificities addressing precisely defined bacterial surface structures.

## Conclusions

In this work, we have developed a rapid test of O-antigen composition in *Salmonella* using bacteriophage tailspike proteins (TSP) as probes, the ELISA-like tailspike adsorption (ELITA) assay. In a microtiter plate format, the ELITA assay could specifically screen which O-antigen serotype is present on cells. TSP are also applicable in culture monitoring by flow cytometry. In contrast to classical type antibodies, TSP could detect phase variable glucosylations on *Salmonella* O-antigens. TSP have large O-antigen binding sites with high binding specificity, moreover, they are highly protease resistant and thermostable proteins. In cases where the O-antigen specificities of TSP are carefully investigated and therefore their binding behavior is exactly defined, we propose to use TSP as robust serotyping proteins in addition to type antibodies and for monitoring O-antigen phase variations in biotechnology applications.

## Methods

### Materials

Lipopolysaccharides (LPS), polysaccharides and oligosaccharides were prepared according to the literature [14, 35]. Phosphate buffered saline (PBS): 16 mM Na_2_HPO_4_, 4 mM KH_2_PO_4_, 115 mM NaCl, pH7.6. TEC-buffer: 50 mM Tris/HCl, 5 mM ethylenediaminetetraacetic acid (EDTA), pH 7.6. PBS-T: PBS buffer supplemented with 200 mM NaCl and 0,2 % (v/v) Tween 20. Flow cytometry (FC) buffer: PBS buffer supplemented with 0.2 % (w/v) bovine serum albumin (BSA). All chemicals were of analytical grade, and ultrapure water was used throughout.

### Tailspike proteins

To N-terminally fuse Strep-tag®II (IBA, Göttingen, Germany), TSP genes were excised from plasmids [16, 36] with restriction enzymes XhoI and BstBI (9NATSP) and NcoI and HindIII (P22TSP) and the fragments ligated into the plasmid pPR-IBA102 (IBA, Göttingen, Germany) and sequenced (GATC Biotech AG, Konstanz, Germany). Expression and purification of 9NA and P22 tailspike proteins (TSP) and mutants followed standard protocols [16, 36].

### Bacterial strains

To initially set up the ELITA-assay, *S*. Typhimurium DB7136 LT2 [37] was used, *E. coli* H TD2158 [38] served as false positive control. All other strains were from the stock culture collection of the National Reference Centre for *Salmonellae* and other *Enterics* at the Robert Koch Institute, Wernigerode, Germany, and are listed in Table 3.

**Table 3:** *Salmonella enterica* subsp. *enterica* strains from the Wernigerode collection used in this study

Strain		Serotype		
		O	H1	H2
13-02615	Paratyphi A	2	a	-
13-02749	Paratyphi A	2	a	-
13-02369	Paratyphi A	1,2	a	-
13-02623	Agona	4	f,g,s	-
13-01155	Brandenburg	4	l,v	e,n,z15
13-03007	Brandenburg	4	l,v	e,n,z15
13-02696	Brandenburg	4	l,v	e,n,z15
13-01714	Bredeney	4	l,v	1,7
13-03023	Brancaster	4	z29	-
13-02636	Chester	4	e,h	e,n,x
13-03043	Derby	4	f,g	-
13-00586	Heidelberg	4	r	1,2
13-03022	Indiana	4	z	1,7
13-02835	Kalamu	4	z4,z24	-
13-01872	Paratyphi B	4	b	1,2
13-02924	Paratyphi B	4	b	1,2
13-01225	Typhimurium	4	i	-
13-02608	Typhimurium	4	i	-
13-02411	Wien	4	b	l,w
13-00008	Abony	4,5	b	e,n,x
13-00308	Heidelberg	4,5	r	1,2
13-01093	Paratyphi B	4,5	b	1,2
13-01583	Paratyphi B	4,5	b	1,2
13-02022	Paratyphi B	4,5	b	1,2
06-01900	Typhimurium	4,5	-	-
13-02786	Typhimurium	5	i	1,2
13-03014	Enteritidis	9	g,m	
13-02939	Eastbourne	9	e,h	1,5
08-01911	Gallinarium	9	-	-
13-02970	Enteritidis	9	g,m	-
13-02971	Enteritidis	9	g,m	-
13-00799	subspecies II	9	l,w	e,n,x
13-03072	Enteritidis	9,12	g,m	-
564	subspecies II	1,9,12,46,27	l,z13,z28	z39
565	subspecies II	1,9,12,46,27	y	z39
13-00188	Anatum	3,10	e,h	1,6
13-00661	Anatum	3,10	e,h	1,6
11-07456	Stendal	11	l,v	1,2
12-05093	Rubislaw	11	r	e,n,x
T4	Typhi	12_1_,12_2_,12_3_	-	-
T2	Typhi	12_1_,12_3_	-	-

### ELISA-like tailspike adsorption (ELITA) assay

Bacteria were cultivated in LB-medium at 37 °C to OD 0.7–1.3. Bacterial suspensions were washed twice with PBS, diluted to OD 0.2 with PBS and adsorbed to multi-well plates over night at 4 °C. Adsorbed bacteria were inactivated with 2 % (w/v) phenol in PBS for 20 min. After three wash steps, surfaces were saturated with 2 % (w/v) BSA in PBS. Adsorbed bacteria were incubated with 0.95 μg per well of Strep-tag® II-TSP in TEC-buffer for 15 min. After washing with PBS-T, bound TSP were detected by adding 0.1 μg ml^-1^ Strep-Tactin® labeled with horseradish peroxidase (IBA, Göttingen, Germany), washed and developed with 1 mg ml^-1^ (w/v) O-Phenylenediamine in 50 mM sodium citrate buffer (pH 6) containing 0.03 % (v/v) H_2_O_2_ for 5 min. The reaction was stopped with 2 M sulfuric acid and the absorbance at 492 nm was measured.

### Matrix-assisted laser-desorption ionization mass spectrometry (MALDI-MS)

Samples were mixed in equal volumes with matrix containing 100 mg ml^-1^ (w/v) 2,5-dihydroxybenzoic acid in 1:1:0.02 (v/v/v) water/acetonitrile/dimethylaniline [39]. Mass spectra were collected on a BrukerMicroflex (Bruker Daltonics, Bremen, Germany) and evaluated with the software mMass [40].

### Capillary electrophoresis

125 μg LPS were incubated with 20 μg mL^−1^ TSP in 10 mM ammonium acetate over night at 37 °C. After precipitation with 90 % (v/v) ethanol the supernatant was collected, dried and dissolved in 1.5 μL 8-aminopyrene-1,3,6-trisulfonic acid (200 mM in 15 % (v/v) acetic acid) and 1.5 μL sodium cyanoborohydride (1 M in tetrahydrofuran), incubated over night at 37 °C and diluted with 97 μL water. Samples were diluted 400-fold and subjected to capillary electrophoresis on a PA-800 (Beckman Coulter) equipped with a LIF detector as described elsewhere [41].

### Flow cytometry

Bacteria were cultivated in LB medium at 37 °C to OD 0.7–1.3, inactivated with 1 % (w/v) phenol and washed with PBS - T. After dilution to OD 0.2 samples were incubated with 5 μg ml^-1^ Strep-tag® II-TSP in PBS for 30 min. After washing with PBS-T, cells were stained with 12.5 μM SytoRed and 125 ng Strep-Tactin®, conjugated to Chromeo 488 (IBA, Göttingen, Germany) in PBS with 0.5 % BSA for one hour and washed. All flow cytometry measurements were carried out on a FACS Calibur instrument equipped with the FlowJo data evaluation software (BD Biosciences, Heidelberg, Germany). Samples were diluted until less than 1000 events per min occurred on the fluorescence channel (λ_ex_: 488 nm/λ_em_: 530 nm). 2 × 10^4^ events per sample in the side scatter channel were collected.

### Bacteriophage assays

Phages were propagated on *S.* Typhimurium DB7136 as described and particles quantified as plaque forming units (pfu) [14, 16]. To determine the number of pfu, bacteria were grown to early log phase in Luria-Bertani (LB) medium at 37 °C, diluted 10^5^ fold in soft agar, inoculated with phage suspensions and spread on LB agar plates. Plaque numbers at different phage dilutions (10^4^ - 10^12^) were counted after incubation at 37 °C overnight.

## Additional files

Additional file 1: Figure S1. HPAEC-PAD analysis of monosaccharide composition of *S*. Brancaster. (PDF 106 kb) Additional file 2: Figure S2. ELITA assay with 44 *Salmonella* strains using 9NA and P22TSP as probes. (PDF 194 kb) Additional file 3: Figure S3. Full flow cytometry data sets of a representative set of *Salmonella* strains from serogroup O4,(5) probed with fluorescently tagged TSP. (PDF 1929 kb)

## Abbreviations

CE-LIF: Capillary electrophoresis with laser-induced fluorescence detection
ELITA: ELISA like tailspike adsorption
HPAEC-PAD: High performance anion exchange with pulsed amperometric detection
LPS: Lipopolysaccharide
MALDI-MS: Matrix-assisted laser-desorption ionization mass spectrometry
TSP: Tailspike protein.
