# Supplementary material for: Bacteriophage tailspike protein based assay to monitor phase variable glucosylations in Salmonella O-antigens
Source: BMC Microbiol. 2016 Sep 7;16(1):207. doi: 10.1186/s12866-016-0826-0 (PMC5015238; doi:10.1186/s12866-016-0826-0)

Supplementary Figure S1

High performance anion exchange chromatography with pulsed amperometric detection (HPAEC-PAD) analysis of monosaccharide composition of *Salmonella* Brancaster O-polysaccharide. To obtain monosaccharides 1 mg of polysaccharide was totally hydrolyzed by incubation with 2N trifluoroacetic acid for 2.5 h at 100 °C.

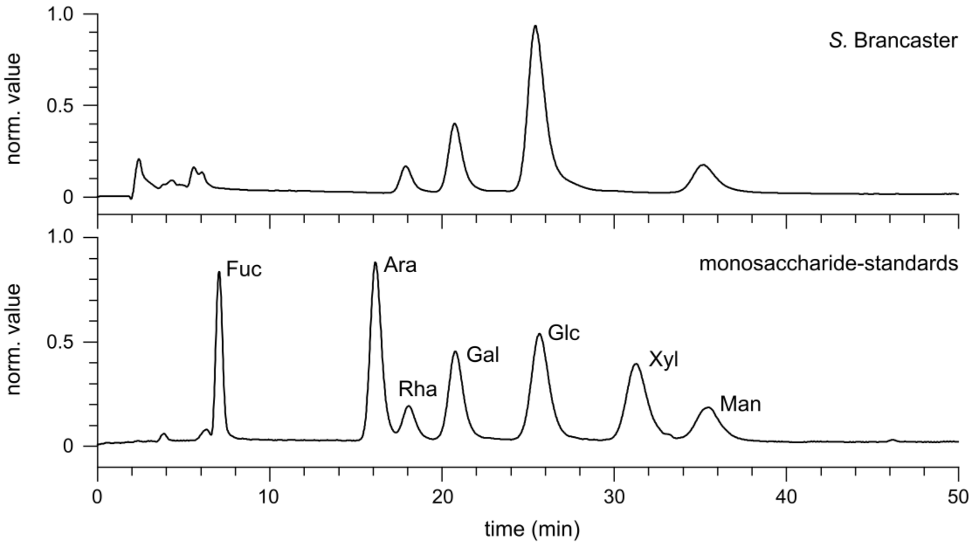

Supplement: Additional file 1: Figure S1. — HPAEC-PAD analysis of monosaccharide composition of S. Brancaster. (PDF 106 kb) [file 12866_2016_826_MOESM1_ESM.pdf]
