# Supplementary material for: Bacteriophage tailspike protein based assay to monitor phase variable glucosylations in Salmonella O-antigens
Source: BMC Microbiol. 2016 Sep 7;16(1):207. doi: 10.1186/s12866-016-0826-0 (PMC5015238; doi:10.1186/s12866-016-0826-0)

Supplementary Figure S2

Identification of *Salmonella* strains with binding O-antigen variants by ELISA-like tailspike adsorption (ELITA) assay using either *Strep*-tag®II-labelled 9NATSP or P22TSP as probes. Error bars represent standard deviation from four samples per strain.

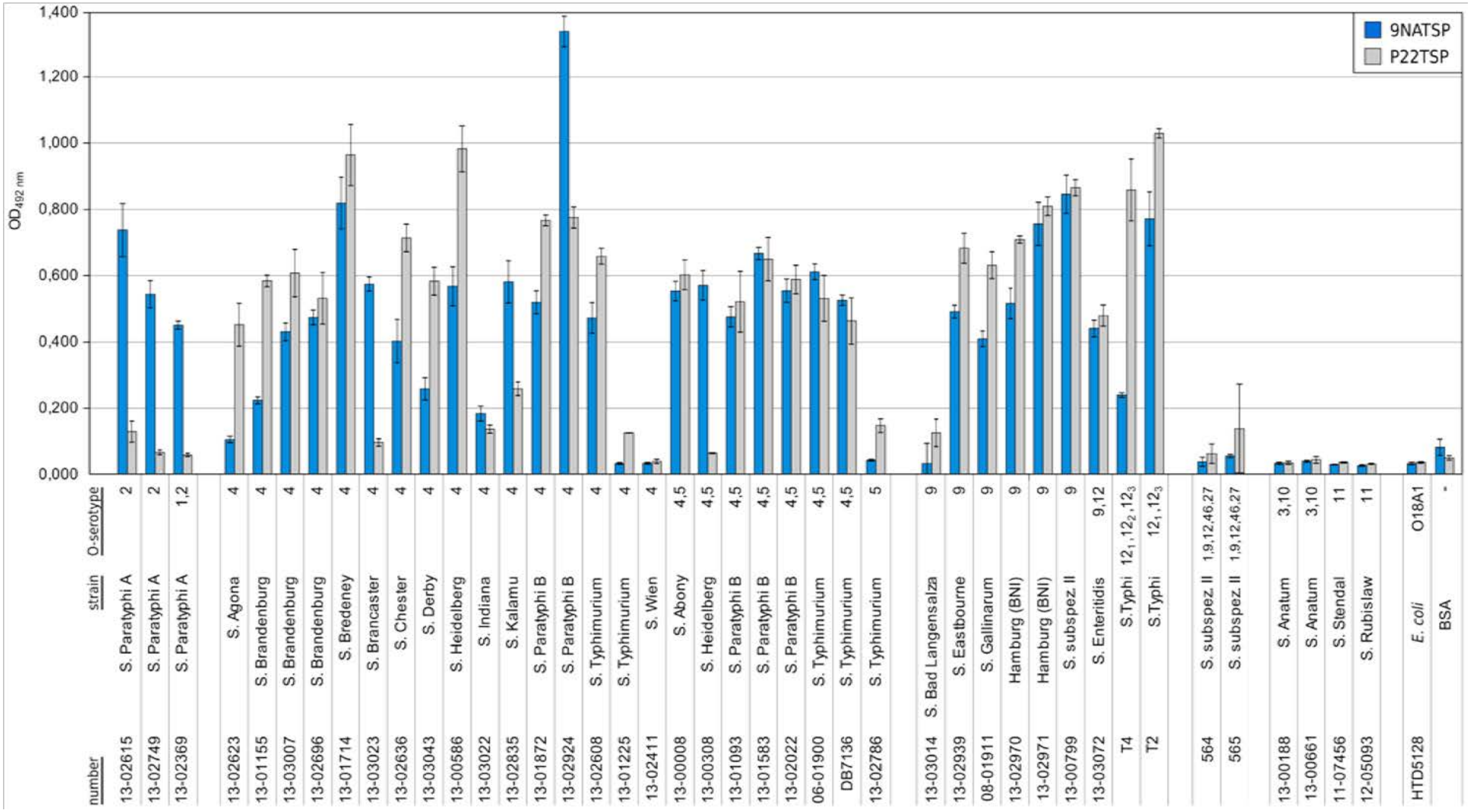

Supplement: Additional file 2: Figure S2. — ELITA assay with 44 Salmonella strains using 9NA and P22TSP as probes. (PDF 194 kb) [file 12866_2016_826_MOESM2_ESM.pdf]
