# Supplementary material for: Bacteriophage tailspike protein based assay to monitor phase variable glucosylations in Salmonella O-antigens
Source: BMC Microbiol. 2016 Sep 7;16(1):207. doi: 10.1186/s12866-016-0826-0 (PMC5015238; doi:10.1186/s12866-016-0826-0)
Supplement: Additional file 3: Figure S3. — Full flow cytometry data sets of a representative set of Salmonella strains from serogroup O4,(5) probed with fluorescently tagged TSP. (PDF 1929 kb) [file 12866_2016_826_MOESM3_ESM.pdf]

Supplementary Figure S3

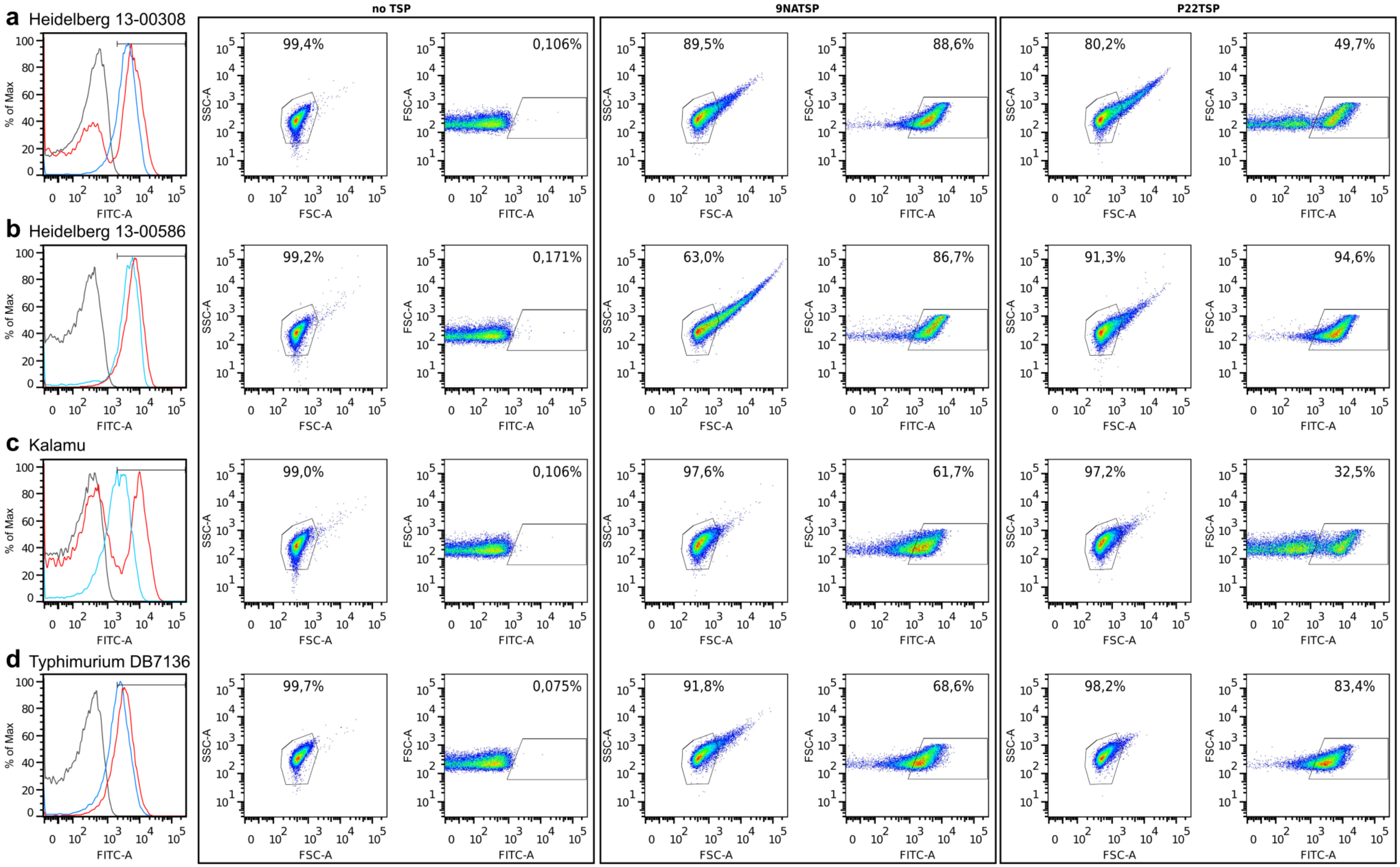

Full flow cytometry data sets of a representative set of *Salmonella* strains from serogroup O4(,5) probed with fluorescently tagged TSP. Flow cytometry data were evaluated by applying two gates. First gate was defined in FSC vs. SSC scatter plot to contain non-aggregated cells, as deduced from the signal intensity in FSC-channel. Second gate in the FITC-A vs. SSC scatter plot contains TSP-bound bacteria. This gate was defined to span signals which showed a higher intensity in FITC-A-channel than control samples without TSP ("no TSP"-box). These gates were applied to samples exposed to a TSP-probe ("9NATSP" and "P22TSP" boxes).

Supplementary Figure S3 (continued)

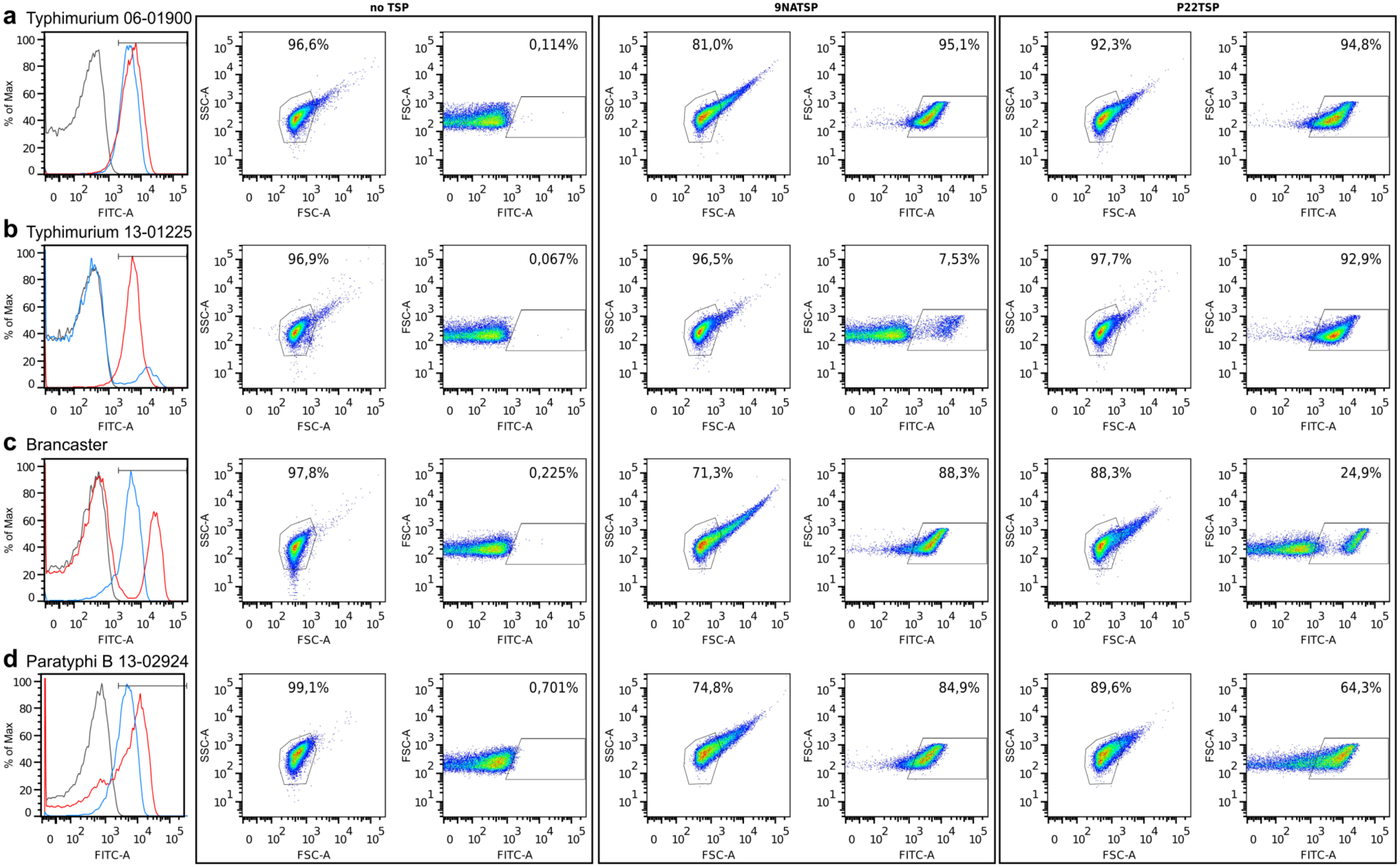

Full flow cytometry data sets of a representative set of *Salmonella* strains from serogroup O4(,5) probed with fluorescently tagged TSP. Flow cytometry data were evaluated by applying two gates. First gate was defined in FSC vs. SSC scatter plot to contain non-aggregated cells, as deduced from the signal intensity in FSC-channel. Second gate in the FITC-A vs. SSC scatter plot contains TSP-bound bacteria. This gate was defined to span signals which showed a higher intensity in FITC-A-channel than control samples without TSP ("no TSP"-box). These gates were applied to samples exposed to a TSP-probe ("9NATSP" and "P22TSP" boxes).
